# Recent progress on BFT in the era of blockchains

**DOI:** 10.1093/nsr/nwac132

**Published:** 2022-07-07

**Authors:** Sisi Duan, Haibin Zhang

**Affiliations:** Institute for Advanced Study, BNRist, and National Financial Cryptography Research Center, Tsinghua University, China; Research Institute of Multidisciplinary Sciences and School of Cyberspace Science and Technology, Beijing Institute of Technology, China

## Abstract

This perspective highlights some recent progress on the research of Byzantine fault tolerant (BFT) consensus protocol in the era of blockchains, including both partially synchronous BFT and asynchronous BFT protocols, their fundamental building blocks, and their variants.

Blockchains can be generally divided into permissionless blockchains (e.g. Bitcoin, Ethereum) and permissioned blockchains (e.g. Hyperledger Fabric, Diem). Byzantine fault tolerance (BFT), which can tolerate Byzantine failures and malicious attacks, has long been understood as a generic building block for critical infrastructures. Nowadays, BFT is known as the standard model for permissioned blockchains. Moreover, BFT is widely and increasingly used in various permissionless blockchains to provide high throughput and transaction finality (e.g. [1]). From a technical perspective, BFT is a generic software technique used for ordering transactions on a distributed system even if a fraction of the nodes are controlled by a malicious adversary.

**Two types of BFT protocols.** Depending on timing assumptions, BFT protocols can be divided into partially synchronous ones (where messages are guaranteed to be delivered within a time bound, but the bound may be unknown) and asynchronous ones (no timing assumption). Both types of protocol do not violate safety. Partially synchronous BFT achieves liveness only when the network environment is synchronous. Asynchronous BFT, however, is typically more complex and less efficient, relying on randomization to achieve probabilistic liveness without assuming synchrony.

**Partially synchronous BFT.** Because of their impressive efficiency, partially synchronous BFT protocols have been widely deployed in the wild. Here we discuss two representative, partially synchronous BFT protocols, belonging to all-to-all BFT and linear BFT, respectively.

*All-to-all BFT.* All-to-all BFT protocols involve all-to-all communication, thus incurring *O*(*n*^2^) message complexity. The classic PBFT protocol falls into this category and is the first BFT protocol without using public-key cryptography [2]. Thus far, it has remained one of the most studied, robust and recognized BFT protocols.

*BFT with linearity.* Being the first BFT protocol with linear communication and enjoying better scalability than PBFT, HotStuff [3] has received significant attention. HotStuff has three phases for both normal-case operation and view change (leader election or rotation), but the known optimal number of phases is two. Thus, follow-up protocols study how to reduce the number of phases. These protocols, however, sacrifice at least one thing for another, e.g. having *O*(*n*^2^) communication for view changes. A recent protocol, Marlin [4], is the first known linear BFT protocol with two phases for normal-case operations and two or three phases for view changes. To achieve the goal, Marlin develops new techniques in designing linear BFT protocols.

Besides the two types of BFT, there are some other protocols such as *chain-based BFT* that organizes replicas in a chain [5,6] and leverages the pipelining message pattern for high throughput.

**Asynchronous BFT.** Different from partially synchronous BFT protocols, asynchronous BFT protocols are leaderless and randomized. Efficient asynchronous BFT protocols may be roughly divided into two categories: the Ben-Or, Kelmer and Rabin (BKR) paradigm [7], including HoneyBadgerBFT, BEAT and EPIC, and the Cachin, Kusawe, Petzold and Shoup (CKPS) paradigm [8], including SINTRA and Dumbo. The BKR paradigm achieves information-theoretic security (using an information-theoretic common coin protocol) and achieves quantum safety (but not quantum liveness); it has *O*(log *n*) running time. In contrast, the CKPS paradigm is only computationally secure and uses less well-established cryptographic pairing assumptions; it has *O*(1) running time but a very large hidden constant.

BKR and its descendants rely on Byzantine reliable broadcast (BRB) and asynchronous binary agreement (ABA). However, BKR does not allow all ABA instances to run in parallel, addressing a well-known performance bottleneck. Zhang and Duan recently proposed PACE [9], a generic framework that removes the bottleneck, allowing fully parallelizable ABA instances. PACE uses BRB and reproposable ABA (RABA), which in contrast to conventional ABA, allows a replica to ‘vote twice.’ The authors demonstrated that by removing this bottleneck, all PACE instantiations, under both failure-free and failure scenarios, drastically outperform existing BFT protocols.

Recently, the work of Narwhal and Tusk offers an innovative approach that provides impressive performance by separating data dissemination from consensus [10]. The approach seems to be compatible with (almost) all BFT protocols.

**Building blocks for BFT.** As discussed above, ABA and BRB are two fundamentally important building blocks for BFT protocols (and in general fault-tolerant-distributed computing). Improved ABA or BRB protocols would lead to more efficient BFT protocols. ABA is a basic consensus primitive, allowing replicas to agree on a common binary value. BRB is a data transmission primitive, allowing correct replicas to receive data reliably. For ABA protocols, PACE provides a comprehensive survey and also proposes an efficient ABA protocol with only two or three steps per round [9]. For BRB protocols, we have recently witnessed several significant results focusing on reducing the communication complexity of BRB protocols [11].

**Long-lived BFT deployment.** So far, we have discussed BFT protocols that *tolerate* failures. We have not yet discussed what one should do when replicas crash or need maintenance. In this scenario, recovering the faulty replicas may not be the best strategy because recovering replicas takes time and it may not even be possible to recover them due to permanent failures. An alternative and sometimes better approach is to allow a new replica to join the system to replace the faulty replica.

In light of the need above, dynamic BFT allowing replicas to join and leave the system dynamically has recently and formally been studied by Duan and Zhang [12]. Duan and Zhang offer various security definitions for dynamic BFT. They also design and implement Dyno, a highly efficient dynamic BFT protocol that can seamlessly take care of membership requests without incurring efficiency degradation.
